# The Molecular Mechanism of the Formation of Four-Membered Cyclic Nitronates and Their Retro (3 + 2) Cycloaddition: A DFT Mechanistic Study

**DOI:** 10.3390/molecules26164786

**Published:** 2021-08-07

**Authors:** Agnieszka Kącka-Zych

**Affiliations:** Faculty of Chemical Engineering and Technology, Cracow University of Technology, Warszawska 24, 31-155 Cracow, Poland; agnieszka.kacka-zych@pk.edu.pl

**Keywords:** four-membered cyclic nitronates, molecular mechanism, density functional theory, Molecular Electron Density Theory

## Abstract

In the present work, the formation of the four-membered cyclic nitronates and the retro (3 + 2) cycloaddition (retro-32CA) reaction of the 4H-[1,2]oxazete 2-oxide were studied using the density functional theory method at the MPWB1K/6-311G(d,p) theoretical level. The electronic structure of 3-tert-butyl-4,4-dimethyl-1,2-dinitro-pent-2-ene was known through electron localization function analysis, natural population analysis, and molecular electrostatic potential analysis. The formation of 4,4-di-tert-butyl-3-nitromethyl-4H-[1,2]oxazete 2-oxide proceeds through a one-step mechanism. The mechanism of the retro-32CA leading to di-tert-butyl ketone and nitrile oxide derivative should be described as an asynchronous two-stage one-step process. The bonding evolution theory study was carried out to clarify the mechanisms of the formation of 4H-[1,2]oxazete 2-oxide and their retro-32CA.

## 1. Introduction

Cyclic nitronates are an important class of compounds because they pose useful intermediates in the synthesis of complex nitrogen-containing scaffolds due to their high reactivity as 1,3-dipoles and availability from nitroalkenes [1,2,3,4]. The main methods of obtaining cyclic four-membered nitronates are thermal and photochemical methods involving the cleavage of unsaturated nitro compounds [5,6,7,8,9].

**Scheme 1:**
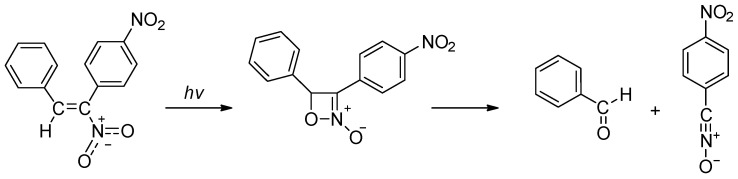
Photochemical reaction described by Pinhey and Rizzardo.

*Pinhey* and *Rizzardo* presented studies on the photochemistry of α,β-unsaturated nitro compounds [5]. They isolated the four-membered cyclic nitronates derivatives starting with cis-α,4-dinitrostilbene, which then breaks down to give benzaldehyde and p-nitrobenzonitrile oxide (Scheme 1). Nevertheless, the Authors do not present specific data confirming the structure of the obtained nitronate.

**Scheme 2:**
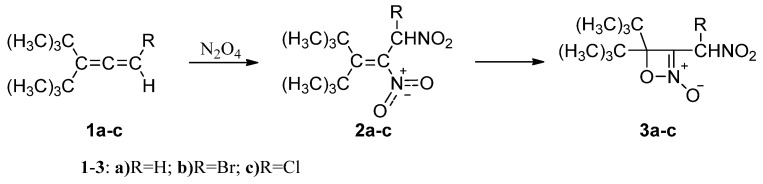
Reaction of 1,1-di-tertbutylallenes (**1a**-**c**) with N_2_O_4_ to the 4H-[1,2]oxazete 2-oxide (**3a**-**c**) derivatives.

*Wieser* and *Berndt* introduced the thermal methods involving the cleavage of unsaturated nitro compounds leading to four-membered cyclic nitronates [6]. In the first step they received 3-substituted 4,4-di-tert-butyl-4H-1,2-oxazete N-oxides (**2a**-**c**) from a reaction of 1,1-di-tertbutylallenes (**1a**-**c**) with dinitrogen tetroxide. The authors say that the addition of N_2_O_4_ to 1,1-di-tertbutylallenes (**1a**-**c**) leads directly to the readily crystallizing oxazete N-oxides (**3a**-**c**) already during work-up at room temperature (Scheme 2). Four-membered cyclic nitronates were obtained with 35–77% yields and confirmed by IR and ^1^H NMR analysis.

**Scheme 3:**
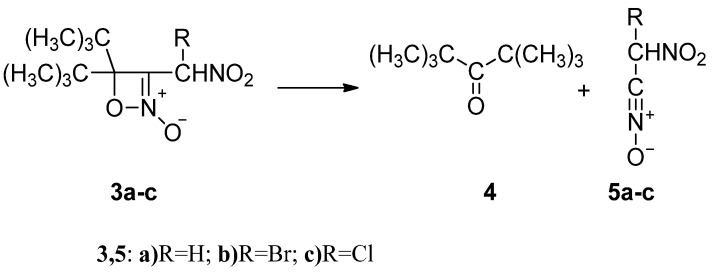
Possible retro-32CA reaction of the 4H-[1,2]oxazete 2-oxide (**3a**-**c**) derivatives.

In my opinion a further retro [3 + 2] cycloaddition (retro-32CA) reaction of 4H-[1,2] oxazete 2-oxide (**3a**-**c**) derivative is possible (Scheme 3). The authors have made no further attempts to explore this possible pathway; therefore, studies to explain the formation of 4H-[1,2] oxazete 2-oxide (**3a**-**c**) derivative and then possible retro-32CA reaction are necessary.

## 2. Results and Discussion

The present theoretical studies explaining the molecular mechanism of the formation of 4,4-di-tert-butyl-3-nitromethyl-4H-[1,2]oxazete 2-oxide (**3a**) and their retro-32CA reaction comprises three different parts: (i) in Section 2.1, an Electron Localization Function (ELF) [10] topological analysis, natural population analysis (NPA) [11,12] and the molecular electrostatic potential (MEP) [13,14] analysis of the 3-tert-butyl-4,4-dimethyl-1,2-dinitro-pent-2-ene (**2a**) is performed in order to characterize the electronic structure of the reagent; (ii) in Section 2.2, the reaction path associated with the formation of **3a** and retro-32CA are studied; and finally, (iii) in Section 2.3, a Bonding Evolution Theory (BET) [15] study associated with the formation of 4,4-di-tert-butyl-3-nitromethyl-4H-[1,2]oxazete 2-oxide (**3a**) and retro-32CA reaction leading to di-tert-butyl ketone (**4**) and nitrile oxide derivative (**5a**) is performed, which portrays bond forming/breaking and enables to understand the mechanistic aspects of these reactions.

### 2.1. Analysis of the Electronic Structure of the 3-Tert-butyl-4,4-dimethyl-1,2-dinitro-pent-2-ene (***2a***)

**Figure 1:**
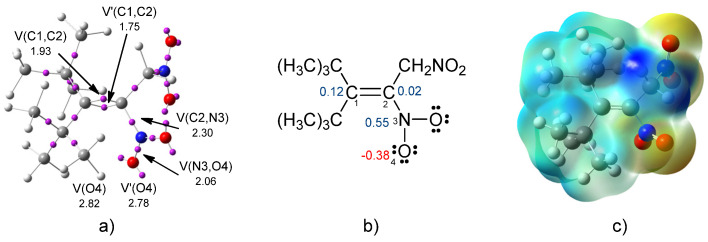
(**a**) The ELF basin attractor positions, together with the most important valence basin populations; (**b**) the proposed ELF-based Lewis-like structures, together with the natural atomic charges, obtained through an NPA; (**c**) and MEP of 3-tert-butyl-4,4-dimethyl-1,2-dinitro-pent-2-ene (**2a**).

In order to understand the electronic structure of 3-tert-butyl-4,4-dimethyl-1,2-dinitro-pent-2-ene (**2a**), we decided to perform a topological analysis of the ELF. The ELF analysis is one appealing procedure that provides a straightforward connection between the electron density distribution and the chemical structure. Therefore, in order to characterize the electronic structure of reagent **2a**, a topological analysis of the ELF was first performed. The ELF basin attractor positions, together with the most important valence basin populations, the ELF-based Lewis structure, together with the natural atomic charges obtained through the NPA analysis and the analysis of the MEP of the substrate, are given in Figure 1. These analyses show us a direct link between the distribution of electron density and the chemical structure, the most important valence basin populations, and the charge distribution in the 3-tert-butyl-4,4-dimethyl-1,2-dinitro-pent-2-ene (**2a**).

ELF topological analysis of the 3-tert-butyl-4,4-dimethyl-1,2-dinitro-pent-2-ene (**2a**), in the most significant region, shows the presence of two V(C1,C2) and V’(C1,C2) disynaptic basins with a population of 1.93 e and 1.75 e, respectively, portraying a double bond character for this region. The C2-N3 and N3-O4 bonding regions in **2a** are characterized by the presence of V(C2,N3) and V(N3,O4) disynaptic basins with a population of 2.30 e and 2.06 e, respectively. The presence of two V(O4) and V’(O4) monosynaptic basins integrating a total population of 5.6 e suggests the presence of three nonbonding electron pairs on the O4 oxygen atom (Figure 1a). Charge distribution of the 3-tert-butyl-4,4-dimethyl-1,2-dinitro-pent-2-ene (**2a**) was examined through the NPA. This analysis indicates that the negative charge is located at the O4 oxygen atom of **2a** (−0.38 e), the C1 and C2 carbon atoms display a negligible positive chare (0.12 e and 0.02 e, respectively), but the N3 nitrogen atom is positively charged by 0.55 e (see Figure 1b). MEP analysis of surface localized the positive charged electrostatic potential (in blue) in the vicinity of the hydrogen atoms of the groups of -CH_2_-NO_2_. In turn, the negative electrostatic potential (in red) is found around the O4 oxygen atom as well as within the rest of the oxygen atoms derived from the nitro groups (Figure 1c).

### 2.2. The Energy Profile Study of the Formation of 4H-[1,2]Oxazete 2-Oxide Derivative (***3a**-**c***) and Their Retro-32CA Reaction

**Figure 2:**
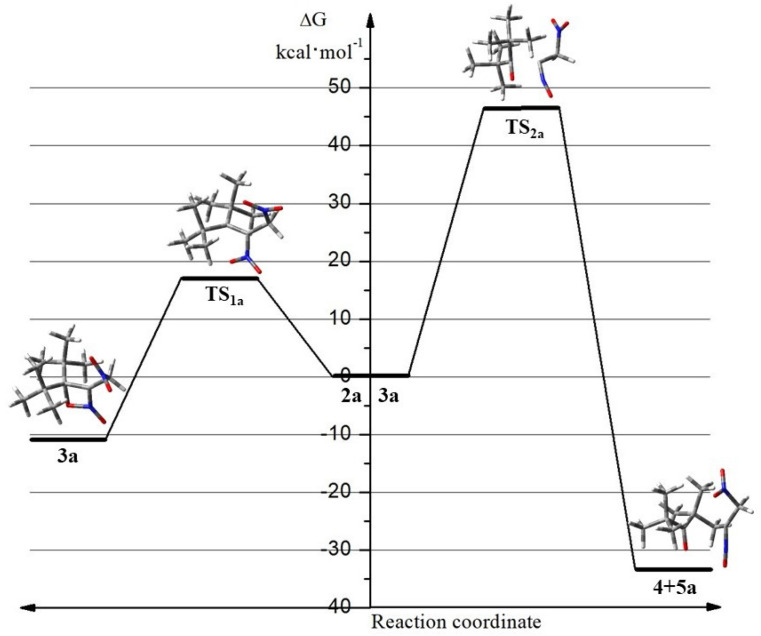
MPWB1K/6-311G(d,p) Gibbs free energy profile (ΔG, kcal·mol^−1^) for the formation of 4-di-tert-butyl-3-nitromethyl-4H-[1,2]oxazete 2-oxide (**3a**) (left side) and retro-32CA reaction of the **3a** (right side).

The relative enthalpies (ΔH), Gibbs free energy (ΔG), and entropies (ΔS) for the stationary points involved in the formation of [1,2]oxazete 2-oxide derivative (**3a**-**c**) and their retro-32CA reaction are collected in Table 1 and Figure 2. The MPWB1K/6-311G(d,p) calculations indicate that formation of 4,4-di-tert-butyl-3-nitromethyl-4H-[1,2]oxazete 2-oxide (**3a**) runs according to a one-step mechanism (Figure 2). The Gibbs free energy value for the transition state (**TS_1a_**) is 17.1 kcal·mol^−1^. Subsequently, the **TS_1a_** is converted to product **3a**. Quantum-chemical calculations using other theory levels show the same process flow. However, calculations with the M06-2X functional indicate higher Gibbs free energies (ΔG = 20.7 kcal·mol^−1^), while the calculations with the B3LYP functional indicate an easier process flow (ΔG = 15.5 kcal·mol^−1^) (Table 1). In the next step, we analyzed the influence of the nature of the substituent on the reaction course. It was found that Cl and Br substituents stimulate the decrease in the Gibbs free energy to **TS_1b_** and **TS_1c_** 9.9 kcal·mol^−1^ and 10.1 kcal·mol^−1^, respectively.

**Figure 3:**
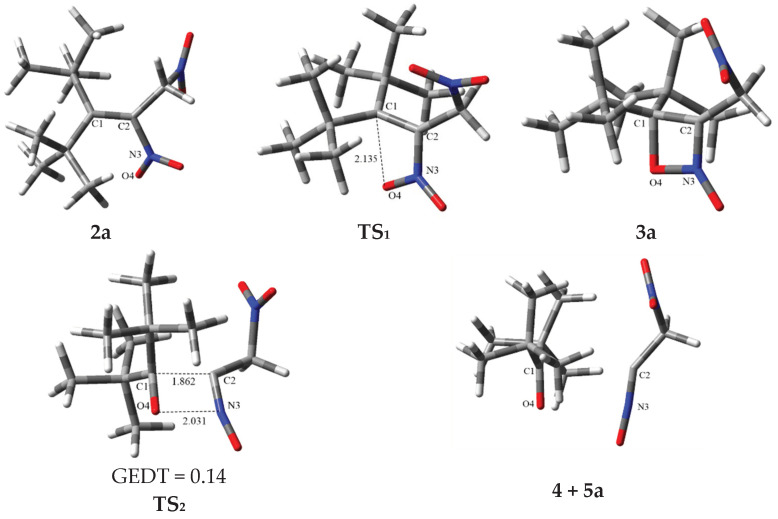
MPWB1K/6-311G(d,p) geometries of the critical structures for formation of 4,4-di-tert-butyl-3-nitromethyl-4H-[1,2]oxazete 2-oxide (**3a**) and retro-32CA reaction leading to di-tert-butyl ketone (**4**) and nitrile oxide derivative (**5a**). Distances are given in angstroms, Å and GEDT in e.

The retro-32CA reaction of 4,4-di-tert-butyl-3-nitromethyl-4H-[1,2]oxazete 2-oxide (**3a**) runs according to an asynchronous one-step mechanism. This process requires a large amount of Gibbs free energy (46.5 kcal·mol^−1^). Additionally, in the case of the retro-32CA reaction, calculations using the M06-2X functional indicate slightly lower Gibbs free energies (ΔG = 43.7 kcal·mol^−1^), while calculations using the B3LYP functional indicate much lower Gibbs free energies (ΔG = 38.2 kcal·mol^−1^). Exploration of the influence of the nature of the substituent on the reaction course showed the same course of the process. The Gibbs free energy remains at the same level for **3b**, while for **3c**, it increases slightly (Table 1). The presented theoretical approach is fully consistent with the experimental work conducted and investigated by *Wieser* and *Berndt* [6]. The GEDT of **TS_2a_** is 0.14 e. In the **TS_2a_**, we observe the rupture of the C1-C2 and N3-O4 single bonds. The C1-C2 and N3-O4 length is 1.862 Å and 2.031 Å, respectively (Figure 3).

### 2.3. BET Study of the Formation of 4,4-Di-tert-butyl-3-nitromethyl-4H-[1,2]oxazete 2-Oxide (***3a***) and Retro-32CA Reaction Leading to Di-Tert-Butyl Ketone (***4***) and Nitrile Oxide Derivative (***5a***)

**Figure 4:**
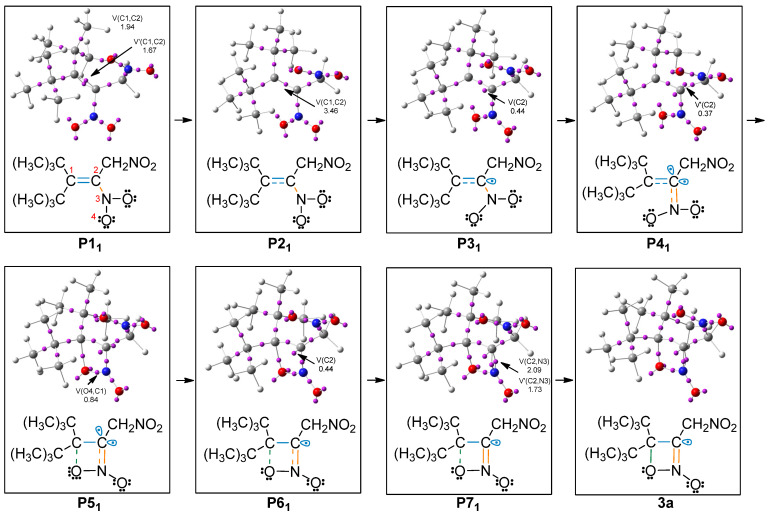
Attractor positions of the most important ELF valence basins of the IRC structures **P1_1_**–**3a** together with simplified representation of the molecular mechanism by Lewis-like structures arising from the topological analysis of the ELF along the reaction path for the formation of 4,4-di-tert-butyl-3-nitromethyl-4H-[1,2]oxazete 2-oxide (**3a**). Figure shows the most important ELF valence basin populations to illustrate which populations disappear and which form in the individual phase. The electron populations, in average number of electrons, is given in e.

In order to explain the changes that occur during the formation of the 4,4-di-tert-butyl-3-nitromethyl-4H-[1,2]oxazete 2-oxide (**3a**), an ELF topological analysis of some selected points on the IRC path was performed. The BET study of this reaction indicates that this reaction is topologically characterized by seven different phases. Figure 4 shows the molecular mechanism represented by Lewis-like structures which result from the ELF topology, while in Table 2, the populations of the most significant valence basins of selected structures of the IRC, further other important parameters are shown.

*Phase I*, 2.774 Å ≥ d(O4-C1) > 2.722 Å, begins at **P1_1_**, which is the first point over the IRC profile. The ELF valence shapes of this point highly resemble those found for the starting reagent (see Table 2) expect little changes in electron populations. At point **P1_1_**, we observed a slight increase in the value of the V(C1,C2) and V(C2,N3) disynaptic and V(O4) and V’(O4) monosynaptic basins. In turn, in the case of V’(C1,C2) and V(N3,O4) disynaptic basins, we observed a slight decline in the electron population values.

*Phase II*, 2.722 Å ≥ d(O4-C1) > 2.309 Å, starts at **P2_1_**. At this point, the two V(C1,C2) and V’(C1,C2) disynaptic basins present at **P1_1_** have merged into a new V(C1,C2) disynaptic basin integrating 3.46 e (Table 2). This change is related to the rupture of the double bond located between C1 and C2 carbon atoms.

*Phase III*, 2.309 Å ≥ d(O4-C1) > 2.266 Å, begins at **P3_1_**, which is associated with the first most relevant change along with the formation of 4,4-di-tert-butyl-3-nitromethyl-4H-[1,2]oxazete 2-oxide (**3a**). In this phase, we observe the formation of a new V(C2) monosynaptic basin integrating 0.44 e. The electron density of the V(C2) monosynaptic basin comes entirely from the strong depopulation of the V(C1,C2) disynaptic basin experienced at **P3_1_** to 3.04 e. This topological change is connected with the formation of the C2 *pseudoradical* center (Figure 4).

*Phase IV*, 2.266 Å ≥ d(O4-C1) > 1.750 Å, begins at **P4_1_**, which is associated with the next important change assuming the formation of a new V’(C2) monosynaptic basin with a population of 0.37 e. We observed the creation of the second *pseudoradical* center at the C2 carbon atom as a result of the depopulation of the V(C1,C2) disynaptic basin. Additionally, in this phase, we find a transition state (**TS_1a_**, d(O4-C1) = 2.146 Å) of the formation of 4,4-di-tert-butyl-3-nitromethyl-4H-[1,2]oxazete 2-oxide (**3a**), which is bonded with the high energy cost of 20.6 kcal·mol^−1^.

*Phase V*, 1.750 Å ≥ d(O4-C1) > 1.628 Å, starts at **P5_1_**. At this point, the next most relevant change along the IRC path takes place: the new V(O4,C1) disynaptic basin is formed with an initial population of 0.84 e (see **P5_1_** in Figure 4). Formation of this disynaptic basin is associated with depopulation of V(C1,C2) disynaptic, V(O4), and V’(O4) monosynaptic basins (Table 2). This change is associated with the formation of an O4-C1 single bond at a distance of 1.75 Å.

*Phase VI*, 1.750 Å ≥ d(O4-C1) > 1.499 Å, begins at **P6_1_**. At this point, the V’(C2) monosynaptic basin presented in the previous point has disappeared. As a result of this change, we observed an increase in the value of the V(C2,N3) disynaptic basin integrating 3.45 e.

*Phase VII*, 1.499 Å ≥ d(O4-C1) > 1.492 Å, starts at **P7_1_** and ends at product **3a**. At this point, the fourth most relevant change takes place with the formation of a new V’(C2,N3) disynaptic basin integrating 1.73 e. This change is associated with the formation of a C2-N3 double bond in final product **3a**.

**Figure 5:**
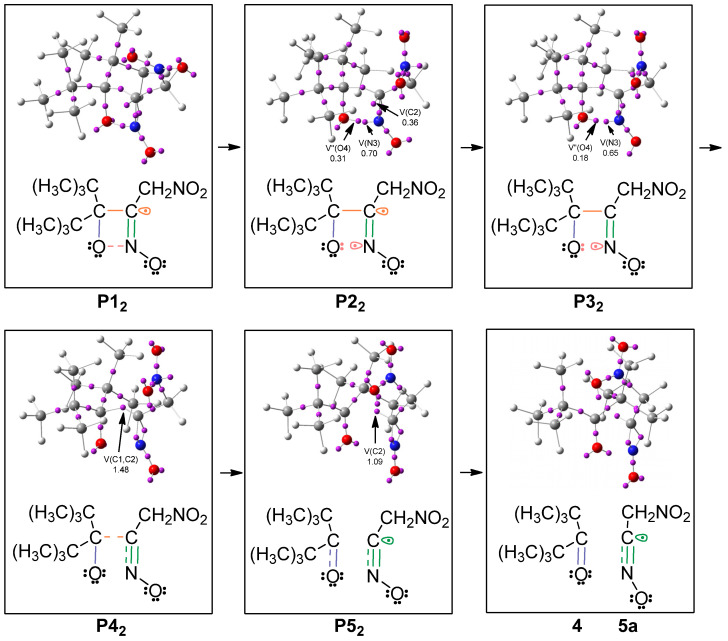
Attractor positions of the most important ELF valence basins of the IRC structures **P1_2–_4** + **5a** together with simplified representation of the molecular mechanism by Lewis-like structures arising from the topological analysis of the ELF along the reaction path for the retro-32CA of the 4,4-di-tert-butyl-3-nitromethyl-4H-[1,2]oxazete 2-oxide (**3a**) leading to di-tert-butyl ketone (**4**) and nitrile oxide derivative (**5a**). Figure shows the most important ELF valence basin populations to illustrate which populations disappear and which form in the individual phase. The electron populations, in average number of electrons, is given in e.

We also wanted to explain the retro-32CA of the 4,4-di-tert-butyl-3-nitromethyl-4H-[1,2]oxazete 2-oxide (**3a**) leading to di-tert-butyl ketone (**4**) and nitrile oxide derivative (**5a**). For this purpose, an ELF topological analysis of some selected points on the IRC path was performed. The BET study of this reaction indicates that this reaction is topologically characterized by five different phases. Figure 5 shows the molecular mechanism represented by Lewis-like structures as a result of the ELF topology, while in Table 3, the populations of the most significant valence basins of selected structures of the IRC, further other important parameters are shown.

*Phase I*, 1.525 Å ≤ d(C1-C2) < 1.594 Å and 1.417 Å ≤ d(N3-O4) < 1.519 Å, starts at **P1_2_**, which is the first point on the IRC path. The ELF picture of **P1_2_** reminds the picture of the 4,4-di-tert-butyl-3-nitromethyl-4H-[1,2]oxazete 2-oxide (**3a**). We have observed a slight increase in the V(C1,C2), V(C2,N3), V’(C2,N3) and V(O4,C1) disynaptic and V(O4) and V’(O4) monosynaptic basins. In the case of the V(C2) monosynaptic and V(N3,O4) disynaptic basins, we see a slight decrease in electron populations (Table 3).

*Phase II*, 1.594 Å ≤ d(C1-C2) < 1.648 Å and 1.519 Å ≤ d(N3-O4) < 1.624 Å, begins at **P2_2_**, which is associated with the first most relevant change along the reaction path. At this point, the V(N3,O4) disynaptic basin disappears, and the new V(N3) and V’’(O4) monosynaptic basins are created with initial populations of 0.70 e and 0.31 e, respectively (Table 3). This topological change is connected with the rupture of the N3-O4 single bond and the formation of N3 and O4 lone pairs (Figure 5). The breaking of the N3-O4 bond occurs at a distance of 1.52 Å.

*Phase III*, 1.648 Å ≤ d(C1-C2) < 1.829 Å and 1.624 Å ≤ d(N3-O4) < 1.886 Å, starts at **P3_2_**. At this point, the V(C2) monosynaptic basin presented in the previous point has disappeared. As a result of this change, we observed an increase in the value of the V(C2,N3) and V’(C2,N3) disynaptic basins integrating 2.31 e and 2.01 e, respectively. This change is related to the disappearance of the *pseudoradical* center at the C2 atom.

*Phase IV*, 1.829 Å ≤ d(C1-C2) < 2.746 Å and 1.886 Å ≤ d(N3-O4) < 2.651 Å, begins at **P4_2_**. This phase is associated with the disappearance of the V(N3) monosynaptic basin, which consequently is associated with an increase in the population of V(C2,N3) and V’(C2,N3) disynaptic basins. We also observed the merging of V(O4), V’(O4), and V’’(O4) monosynaptic basins into new V(O4) and V’(O4) monosynaptic basins with a total population of 5.72 e. In this phase, we find a transition state (**TS_2a_**, d(C1-C2) = 1.927 Å and d(N3-O4) = 2.011 Å) of the retro-32CA of 4,4-di-tert-butyl-3-nitromethyl-4H-[1,2]oxazete 2-oxide (**3a**), which is connected with a high energy cost of 40.5 kcal·mol^−1^.

The last *Phase V*, 2.746 Å ≤ d(C1-C2) < 2.921 Å and 2.651 Å ≤ d(N3-O4) < 2.723 Å, is located between **P5_2_** and final products 4 and 5a. In this phase, we observed the next significant change along the reaction course. The V(C1,C2) disynaptic basin, present in the previous phase, has disappeared, and the new V(C2) monosynaptic basin is created, integrating 1.09 e. These changes are associated with the rupture of the second C1-C2 single bond and the formation of the C2 *pseudoradical* center.

Considering the BET analysis of the studied formation of 4,4-di-tert-butyl-3-nitromethyl-4H-[1,2]oxazete 2-oxide (**3a**) and retro-32CA reaction leading to di-tert-butyl ketone (**4**) and nitrile oxide derivative (**5a**), we can draw some important conclusions: (i) the molecular mechanism formation of 4,4-di-tert-butyl-3-nitromethyl-4H-[1,2]oxazete 2-oxide (**3a**) can be characterized by seven topologically different phases (Table 2) while the retro-32CA of the 4,4-di-tert-butyl-3-nitromethyl-4H-[1,2]oxazete 2-oxide (**3a**) can be described by five different phases (Table 3); (ii) the molecular mechanism for creating product 3a begins with the rupture of the double bond between C1 and C2 carbon atoms. Then, we observed formation of two *pseudoradical* centers at the C2 carbon atom. In the final stage, we observed the creation of O4-C1 single and C2-N3 double bonds; (iii) In the initial stage of the retro-32CA, we notice the rupture of the first N3-O4 single bond. The next changes are the disappearance of the *pseudoradical* center at the C2 atom and, in the final stage, rupture of the second C1-C2 single bond and formation of the C2 *pseudoradical* center.

## 3. Computational Details

All calculations carried out as part of this work and associated with the formation of 4,4-di-tert-butyl-3-nitromethyl-4H-[1,2]oxazete 2-oxide (**3a**) and their retro-32CA reaction were performed by means of the GAUSSIAN 16 package [16] in the Prometheus computer cluster of the CYFRONET regional computer center in Cracow. The geometries of all reactants, transition state structures (TSs), and products of the reactions were fully optimized using the MPWB1K [17], B3LYP [18,19], and M06-2X [20] functionals together with the 6-311G(d,p) basis set. Stationary points were characterized by frequency calculations. All reactants and products had positive Hessian eigenvalues. All TSs had only one negative eigenvalue in their diagonalized Hessian matrices, and their associated eigenvectors were confirmed to correspond to the motion along the reaction coordinate under consideration. The global electron density transfer (GEDT) [21] values were calculated as the sum of the natural atomic charges (q), obtained by an NPA [11,12], by the equation: GEDT (f) GEDT (f) = Σ_*q*∈*f*_*q*, for all atoms belonging to each fragment (f) of the TSs at the MPWB1K/6-311G(d,p) level of theory. The values of electronic energies, enthalpies, entropies, and Gibbs free energies were calculated with the standard statistical thermodynamics at 1 atm [22].

Electron Localization Function (ELF) [10] studies were performed with the TopMod [23] program using the corresponding MPWB1K/6-311G(d,p) monodeterminantal wavefunctions considering the standard cubical grid of step size of 0.1 Bohr. The molecular mechanism of the formation of 4,4-di-tert-butyl-3-nitromethyl-4H-[1,2]oxazete 2-oxide (**3**) and their retro-32CA reaction was analyzed according to the Bonding Evolution Theory (BET) [15]. For the BET studies, the corresponding reaction paths were followed by performing the topological analysis of the ELF for the formation of 4,4-di-tert-butyl-3-nitromethyl-4H-[1,2]oxazete 2-oxide (**3a**) at least 160 nuclear configurations along the IRC path and for retro-32CA reaction leading to di-tert-butyl ketone (**4**) and nitrile oxide derivative (**5a**) at least 181 nuclear configurations along the IRC path. The ELF molecular geometries and basin attractor positions were visualized using the GaussView program [24]. ELF localization domains were represented by using the Paraview software at an iso value of 0.75 a.u [25,26]. A similar approach, entering in Molecular Electron Density Theory (MEDT) [27], to elucidate the molecular mechanism has been used successfully in various types of reactions. The used computational method has been shown to give results that are in complete agreement with experimental outcomes of previous reactions [28,29,30,31,32,33,34,35].

## 4. Conclusions

The formation of 4H-[1,2]oxazete 2-oxide (**3a–c**) derivatives and their retro-32CA reaction experientially investigated by *Wieser* and *Berndt* [6] was theoretically studied at the MPWB1K/6-311G(d,p) computational level. The formation of 4H-[1,2]oxazete 2-oxide (**3a**) proceeds through a one-step mechanism. The mechanism of the retro-32CA reaction should be described as an asynchronous two-stage one-step process.

The BET analysis of the formation of 4,4-di-tert-butyl-3-nitromethyl-4H-[1,2]oxazete 2-oxide (**3a**) allowed for the distinction of seven topologically different phases connected with the molecular mechanism of this reaction. This reaction begins with the rupture of the double bond between C1 and C2 carbon atoms in a 3a molecule. Subsequently, *Phases III* and *IV* are related to the formation of two *pseudoradical* centers at the C2 carbon atom. In *Phase V*, we observed the formation of the first O4-C1 single bond with an initial population of 0.84 e. In the final stage of this reaction, we notice the formation of the C2-N3 double bond. In turn, the mechanism of retro-32CA reaction has been described by five different phases. This reaction begins with the rupture of the first N3-O4 single bond. In the next step, we observed the stage rupture of the second C1-C2 single bond and the formation of a *pseudoradical* center at the C2 carbon atom. The presented theoretical calculations are fully consistent with the experimental work. So, the proposed scheme should be assumed to be general for the formation of four-membered cyclic nitronates and their transformation.

## Figures and Tables

**Table 1:** The relative electronic energies, enthalpies, Gibbs free energy (ΔE, ΔH, and ΔG, in kcal·mol^−1^) and entropies (ΔS, in cal·mol^−1^·K^−1^), calculated at different functionals and 6-311G(d,p) basic set, for the stationary points involved in the formation of 4H-[1,2]oxazete 2-oxide derivative (**3a**-**c**) and retro-32CA reaction leading di-tert-butyl ketone (**4**) and nitrile oxide derivative (**5a**-**c**).

R	Functional	Reaction	Thermodynamic Parameters			
			ΔE	ΔH	ΔG	ΔS
H	M06-2X	**2a→TS**_**1a**_	20.8	19.7	20.7	−3.6
		**2a→3a**	−8.8	−8.2	−8.0	−0.6
		**3a→TS**_**2a**_	49.5	44.8	43.7	3.5
		**3a→4 + 5a**	−11.0	−16.1	−33.0	56.6
	B3LYP	**2a→TS**_**1a**_	16.6	14.7	15.5	−2.7
		**2a→3a**	−7.1	−7.7	−7.7	−0.1
		**3a→TS**_**2a**_	42.3	39.6	38.2	4.7
		**3a→4 + 5a**	−27.7	−30.0	−46.3	54.9
	MPWB1K	**2a→TS**_**1a**_	17.7	15.8	17.1	−4.3
		**2a→3a**	−10.8	−11.5	−10.9	−2.0
		**3a→TS**_**2a**_	50.9	47.9	46.5	4.7
		**3a→4 + 5a**	−13.3	−15.9	−33.2	58.1
Br		**2b→TS**_**1b**_	8.8	9.2	9.9	−2.2
		**2b→3b**	−18.6	−19.0	−19.2	0.9
		**3b→TS**_**2b**_	44.9	47.7	46.5	3.9
		**3b→4 + 5b**	−10.8	−13.5	−31.0	58.5
Cl		**2c→TS**_**1c**_	9.3	9.5	10.1	−2.0
		**2c→3c**	−17.5	−18.1	−18.4	1.0
		**3c→TS**_**2c**_	45.7	48.0	47.5	1.7
		**3c→4 + 5c**	−9.7	−12.4	−30.2	59.5

**Table 2:**
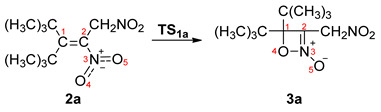
ELF valence basin populations, distances of the forming bonds, MPWB1K/6-311G(d,p) relative ^a^ electronic energies (ΔE) of the IRC structures, **P1**–**3a**, defining the seven different phases characterizing the molecular mechanism of the formation of the 4,4-di-tert-butyl-3-nitromethyl-4H-[1,2]oxazete 2-oxide (**3a**). **2a**, TS_1a_ and product 3a are also included. Distances are given in angstroms, Å, electron populations in average number of electrons, e, and relative energies in kcal·mol^−1^.

Structures	2a	P1_1_	P2_1_		P3_1_		P4_1_		P5_1_		P6_1_		P7_1_		3a	TS_1a_
Phases		*I*		*II*		*III*		*IV*		*V*		*VI*		*VII*		
d(O4-C1)		2.774	2.722		2.309		2.266		1.750		1.628		1.499		1.492	2.146
ΔE^a^		0.0	4.5		10.1		18.6		10.9		2.4		−4.4		−8.1	20.6
V(C1,C2)	1.93	1.94	3.46		3.04		2.62		2.18		2.17		2.15		2.12	2.39
V’(C1,C2)	1.75	1.67														
V(C2)					0.44		0.47		0.48		0.44		0.50		0.51	0.47
V’(C2)							0.37		0.30							0.36
V(C2,N3)	2.30	2.44	2.42		2.51		2.79		3.25		3.45		2.09		2.01	2.98
V’(C2,N3)													1.73		1.79	
V(N3,O4)	2.06	1.98	1.92		1.76		1.76		1.62		1.55		1.52		1.50	1.69
V(O4)	2.82	2.84	2.88		2.91		2.96		2.71		2.46		2.47		2.37	2.94
V’(O4)	2.78	2.81	2.84		2.81		2.89		2.60		2.50		2.43		2.39	2.71
V(O4,C1)									0.84		1.06		1.23		1.54	

^a^ Relative to the first point of IRC, **P1_1_**.

**Table 3:**
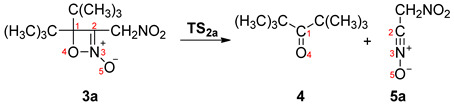
ELF valence basin populations, distances of the forming bonds, MPWB1K/6-311G(d,p) relative ^a^ electronic energies (ΔE) of the IRC structures, **P1_2_**–**4** + **5a**, defining the five different topically phases retro-32CA of the 4,4-di-tert-butyl-3-nitromethyl-4H-[1,2]oxazete 2-oxide (**3a**). **3a**, **TS_2a_**, and products **4** and **5a** are also included. Distances are given in angstroms, Å, electron populations in average number of electrons, e, and relative energies in kcal·mol^−1^.

Structures	3a	P1_2_	P2_2_		P3_2_		P4_2_		P5_2_		4 + 5a	TS_2a_
Phases		*I*		*II*		*III*		*IV*		*V*		
d(C1-C2)		1.525	1.594		1.648		1.829		2.746		2.921	1.927
d(N3-O4)		1.417	1.519		1.624		1.886		2.651		2.723	2.011
ΔE ^a^		0.0	0.6		5.9		32.9		−4.7		−18.3	40.5
V(C1,C2)	2.12	2.13	1.95		1.67		1.48					1.42
V(C2)	0.51	0.43	0.36						1.09		1.26	
V(C2,N3)	2.01	2.02	2.21		2.31		2.59		2.62		2.68	2.60
V’(C2,N3)	1.79	1.83	1.89		2.01		2.26		2.30		2.43	2.27
V(N3,O4)	1.50	1.39										
V(N3)			0.70		0.65							
V(O4)	2.47	2.53	2.68		2.74		2.89		2.68		2.56	2.84
V’(O4)	2.49	2.51	2.53		2.57		2.83		2.63		2.52	2.78
V’’(O4)			0.31		0.18							
V(O4,C1)	1.54	1.64	1.79		1.91		2.32		2.61		2.73	2.43

^a^ Relative to the first point of IRC, **P1_2_**.

## Data Availability

The data presented in this study are available on request from the corresponding author.
